# Clinical and radiological effects of Corticosteroid injection combined with deep transverse friction massage and Mill’s manipulation in lateral epicondylalgia–A prospective, randomized, single-blinded, sham controlled trial

**DOI:** 10.1371/journal.pone.0281206

**Published:** 2023-02-13

**Authors:** Gopal Nambi, Mshari Alghadier, Anju Verma, Osama R. Aldhafian, Naif N. Alshahrani, Ayman K. Saleh, Mohamed A. Omar, Tohamy G. T. Hassan, Mohamed Nagah Ahmed Ibrahim, Hassan Fathy El Behairy

**Affiliations:** 1 Department of Health and Rehabilitation Sciences, College of Applied Medical Sciences, Prince Sattam bin Abdulaziz University, Al Kharj, Saudi Arabia; 2 Faculty of Medicine and Health, Department of Exercise and Sports Sciences, School of Health Sciences, University of Sydney, New South Wales, Australia; 3 Department of Surgery, College of Medicine, Prince Sattam bin Abdulaziz University, Al Kharj, Saudi Arabia; 4 Orthopedic Surgery Department, King Fahad medical city, Ministry of Health, Riyadh, Saudi Arabia; 5 Faculty of Medicine for Girls, Department of Orthopedic Surgery, Al-Azhar University, Cairo, Egypt; 6 Faculty of Medicine for Girls, Al-zhraa University Hospital, Al-Azhar University, Cairo, Egypt; King Khalid University, SAUDI ARABIA

## Abstract

**Background:**

The knowledge about the effective implementation of corticosteroid injection (CS) with deep transverse friction massage (DTFM) and Mill’s manipulation (MM) on clinical and radiological changes (Magnetic resonance imaging—MRI and Ultra sound) in lateral epicondylalgia (LE) is lacking. Therefore, the objective of this study is proposed to find and compare the effects of corticosteroid injection (CS) DTFM and Mill’s manipulation on clinical and radiological changes in lateral epicondylalgia.

**Design, setting, participants:**

Randomized, single-blinded, controlled study was conducted on 60 LE participants at university hospital. The active MM group (n = 30) received corticosteroid injection with DTFM and active Mill’s manipulation (MM) three sessions a week for 4 weeks and the sham MM group received corticosteroid injection with sham manipulation. The primary outcome was pain intensity, measured with the visual analog scale. The other outcome measures were percentage of injury measured by MRI and ultrasound, functional disability, handgrip strength, patient perception, kinesiophobia, depression status and quality of life which were measured at 4 weeks, 8weeks and at 6 months follow up.

**Results:**

The between-group difference in pain intensity at 4 weeks was 1.6 (CI 95% 0.97 to 2.22), which shows improvement in the active group than sham group. The similar effects have been noted after 8 weeks and at 6 months 2.0 (CI 95% 1.66 to 2.33) follow up in pain intensity. Similar improvements were also found on percentage of injury, functional disability, handgrip strength, patient perception, kinesiophobia, depression status and quality of life (p = 0.001).

**Conclusion:**

Corticosteroid injection with DTFM and Mill’s manipulation was superior to sham group for improving pain, percentage of injury, functional disability, handgrip strength, patient perception, kinesiophobia, depression status and quality of life in people with lateral epicondylalgia.

**Trial registration:**

**Clinical trial registration:** CTRI/2020/05/025135 trial registered prospectively on 12/05/2020. https://trialsearch.who.int/Trial2.aspx?TrialID=CTRI/2020/05/025135.

## 1. Introduction

Lateral epicondylalgia (LE), is a common musculoskeletal condition with clinical symptoms of pain and inflammation over the proximal attachment of the wrist extensor tendon of the humerus. It is frequently known as “lateral epicondylitis” or “tennis elbow” and maximum incidences occur between 30 and 65 years of the age [1]. The other symptoms may include stiffness or weakness in the elbow and having difficulties in functional activities of hand such as gripping, holding, pinching etc [2]. It is caused by direct injury or repeated stress or motion of the soft tissues surrounding the elbow joint. It is estimated that LE has an annual incidence of 1–3% in the population and has a significant socioeconomic impact on the society. Different conservative, medical and surgical treatments are available for this condition [3]. Amongst these, corticosteroid injection and physical therapy are widely used treatment and most studies shows the superior effect of corticosteroid injection in LE, [3,4] however contradicted by some studies [5,6]. The poor outcome of corticosteroid injection was due to high recurrence rate (72%) on a long term basis [5]. Administration of corticosteroid locally at the painful region alters the release of endogenous substances and inhibits the formation of collagen fibers, extracellular matrix (ECM) and granulation tissue [7]. However, it has not been clinically proven weather these changes are clinically helpful or harmful to the LE patients.

Recently, corticosteroid injections are discouraged by many physicians because of high recurrence rate [5]. Therefore, orthopedic surgeons usually encourage physical therapy in combination with corticosteroid injection for lateral epicondylalgia. Physical therapy treatments offered for LE include: hydrocolloid packs, infrared radiation, short wave diathermy, ultrasound, laser, physical exercises, manipulation and splint application [3,8]. A clinical commentary by Vicenzino B et al found evidence for the use of elbow manipulation and therapeutic exercise for LE on a short-term basis [9]. Manual therapy and eccentric strength training are the two physiotherapeutic treatment methods that have the greatest beneficial effects in treating LE [10]. In manual therapy both Cyriax approach (Deep transverse friction massage with Mill’s manipulation) and Mulligan mobilization with movement (MWM) decreased pain and improved functional status in lateral epicondylitis patients [11]. A study by Nagrale AV et al investigated that Cyriax approach is a superior treatment approach compared to conventional physiotherapy treatment in managing lateral epicondylalgia. The proposed mechanism of Mill’s manipulation is the lengthening of scar tissue following the rupture of adhesions due to the manipulation. This increased length decreases tension on the scar leading to less pain, effectively converting a tear shaped like a “V” into one resembling a “U”. The resulting gap is filled with fibrous tissue, resulting in permanent lengthening and abolition of pain [12].

Along with the regular investigations, there is a need to find the radiological changes after corticosteroid injection with DTFM and Mill’s manipulation in lateral epicondylalgia. The advanced radio imaging techniques such as magnetic resonance imaging (MRI) and ultrasonography (US) are used to find the extent of disease, the associated problems and measure the degree of soft tissue injury [13,14]. So far no studies have been conducted to find the radiological changes after corticosteroid injection with DTFM and Mill’s manipulation in treating lateral epicondylalgia. Therefore, this study aims to find and compare the clinical and radiological changes after corticosteroid injection with DTFM and Mill’s manipulation versus sham treatment in patients with lateral epicondylalgia by randomized control trial method. Clinical and radiological analysis using MRI and US will provide real time changes in the soft tissues after different interventions, which will help the therapists to select the optimum intervention for lateral epicondylalgia.

## 2. Materials and methods

### 2.1 Trial design

This trial was a prospectively registered, randomized, parallel group, sham controlled trial executed at department of physical therapy, college of applied medical sciences, Prince Sattam bin Abdulaziz University, Saudi Arabia. Participants were recruited between 1^st^ June 2020 and 1^st^ August 2021. This study was designed and conducted in accordance with the principles of the Declaration of Helsinki and it has been approved by the Department Ethics Committee, Prince Sattam bin Abdulaziz University, Saudi Arabia with an ethical approval number RHPT/020/012. The trial was registered prospectively in Clinical trial registry, India with registration number CTRI/2020/05/025135 and registered prospectively on 12/05/2020.

### 2.2 Participants

The participants were invited by sending personal email or mobile call and they were recruited from University hospital and King Khalid hospital, Al Kharj, Saudi Arabia by an orthopedic surgeon. They were informed about the harms and benefits of the research through an information form. Subjects who consented to participate in the study were selected for the study and the written informed consent was obtained from all subjects. Male, aged between 18 and 60 years, with a clinical diagnosis of chronic (> 2 months of pain) lateral epicondylalgia (M77.1 in ICD-10 –International classification of diseases), with pain intensity between 3 to 8 on the visual analog scale (VAS) were allowed to participate. Only male subjects were included in this study due to their responses to the interventions when compared to the female participants, which may alter the study reports. Participants with prior steroid injection therapy, associated neck or arm pain, severe musculoskeletal, neural, somatic and psychiatric conditions, waiting for surgery, having alcohol or drug abuse, involving in other weight training programs, and red flags to manipulation were excluded from the study. Participants with other soft tissue injuries, fracture at the limbs, deformities were also excluded from the study.

**Fig 1:**
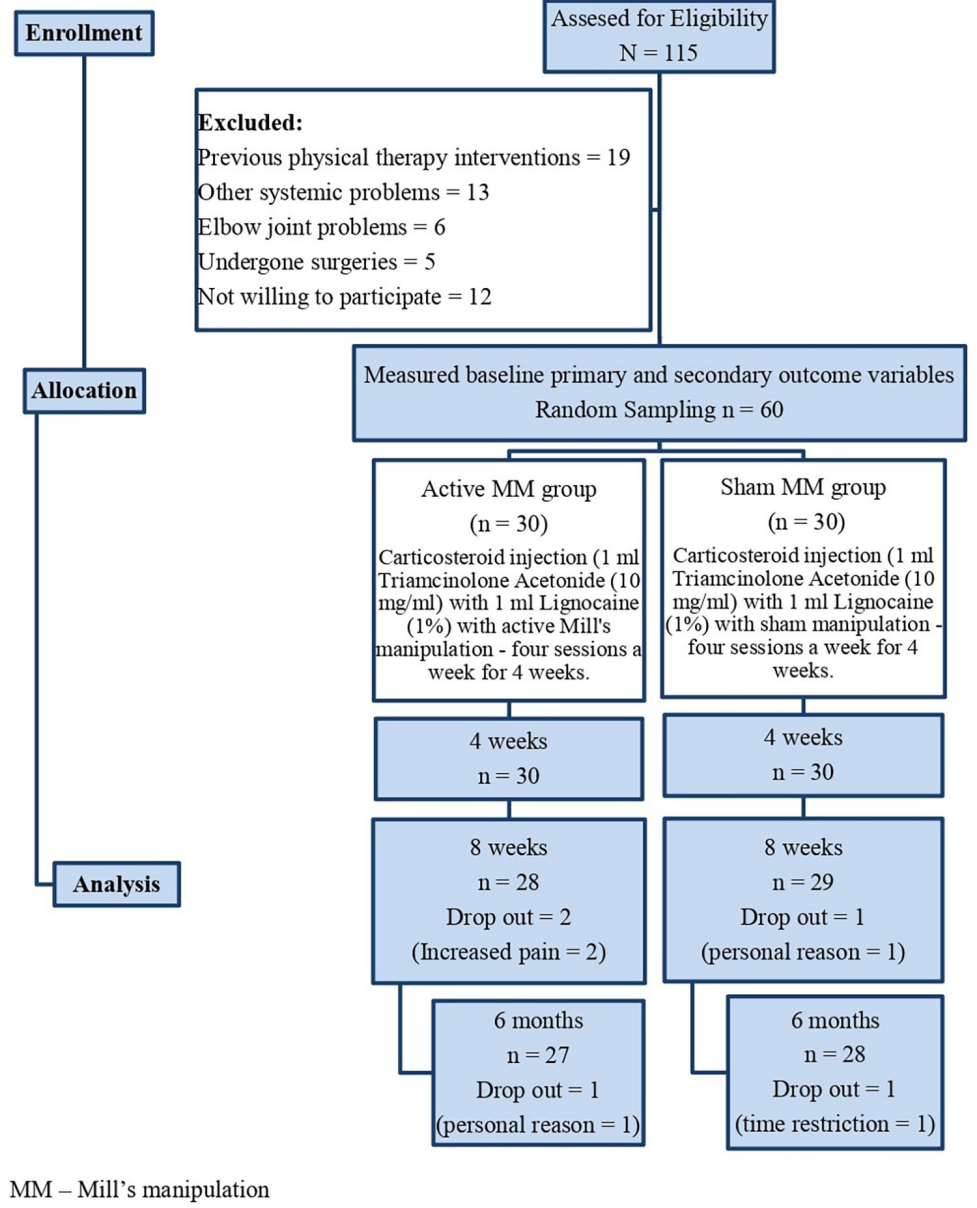
Flow chart showing the study details.

The participants were randomized using computer generated randomization method and allocated into two groups: corticosteroid injection with DTFM and Mill’s manipulation–Active group (n = 30) and Sham group (n = 30) and the flow of study participants was shown in Fig 1. Sealed opaque envelopes were used and allocation was picked by each participant from a sealed opaque envelope, given by the consulting physiotherapist, after eligibility for the study was established. The envelope contained only two pieces of paper. Participants were asked to pick one piece of paper from the envelope. One piece of paper had the letter ‘A’ for active treatment group and the other, letter ‘S’ for sham treatment group. The participant’s group allocation was only informed to the physical therapists who provided the treatments. But, the therapist (blind assessor) who was measuring the outcomes at various intervals was blinded. Also the participants were unaware of the treatment they received (blind participants) and they were instructed not to share the treatment information with the fellow participants. Both groups received the concerned intervention for a period of three sessions per week for 4 weeks. The outcome measures were collected by a blinded physical therapist at baseline, after 4 weeks, 8 weeks and at 6 months follow up. A total of 2 physical therapists with more than 15 years of experience treated the participants.

### 2.3 Interventions

Following corticosteroid injection, the recommended physical therapy was given for 4 weeks, after which the participants were asked to exercise at home for another 4 weeks. The participants maintained an exercise log book during the study period.

#### 2.3.1 Corticosteroid injection

A physical examination was performed by an orthopedic surgeon before the administration of injection. 1 ml Triamcinolone Acetonide (10 mg/ml) (Kenacort- A 10) with 1 ml Lignocaine (1%) was administered into the most palpably tender point in the region of the lateral epicondyle [15]. To maintain participant blinding, the participants were not allowed to see the procedure of administration of injection. In addition, post-injection instructions were given to all the participants. They were asked to take rest and not engage in strenuous activities for one week following injection, even if they experience pain relief. Any adverse consequences were noted and treated by the prime investigator.

#### 2.3.2 Physical therapy

A week after the injection the participants started with the physical therapy interventions. All participants received the respective physical therapy for 3 sessions per week for 4 weeks, each session lasted for 30 to 40 minutes.

All the participants underwent baseline evaluation of variables before the administration of injection. To avoid intervention bias, a fixed physical therapy protocol was prepared on the basis of recent evidence with the objectives of ameliorating pain, increasing functional activity and soft tissue healing. A holistic approach consisting of physical modalities, exercise protocols and patient education were used to obtain these objectives. Participants in active MM group underwent deep transverse friction massage (DTFM) and Mill’s manipulation. Deep transverse friction massage (DTFM) is a specific type of connective tissue massage applied precisely to the soft tissues around the elbow. It is important that the depth of friction applied is tolerable to the patient. It was applied transversely to the specific tissue involved and applied for 5 minutes. This technique is applied to prepare the structures for the manipulation.

For manipulation, the participant was asked to sit comfortably with proper backrest. The treating therapist stands behind the patient and grasp the patient’s arm under the crook of the elbow with the shoulder joint abducted to 90° and medially rotated. Therefore, the patient’s forearm is pronated. Place the thumb of other hand in the web space between the patient’s thumb and index finger and fully flex the patient’s wrist and pronate the forearm. Move the hand supporting the crook of the elbow on to the posterior surface of the elbow joint and, while maintaining full wrist flexion and pronation, extend the patient’s elbow until that all the slack has been taken up in the tendon. Apply a high velocity low amplitude thrust (HVLAT) by side flexing the body away from the arms and pushing downwards with the hand over the patient’s elbow. This procedure is conducted only once at each session because it is an uncomfortable procedure for the patient [16]. For the sham group, low velocity high amplitude movement was performed at the elbow joint, which was not comprehensible to the study participants.

**Fig 2:**
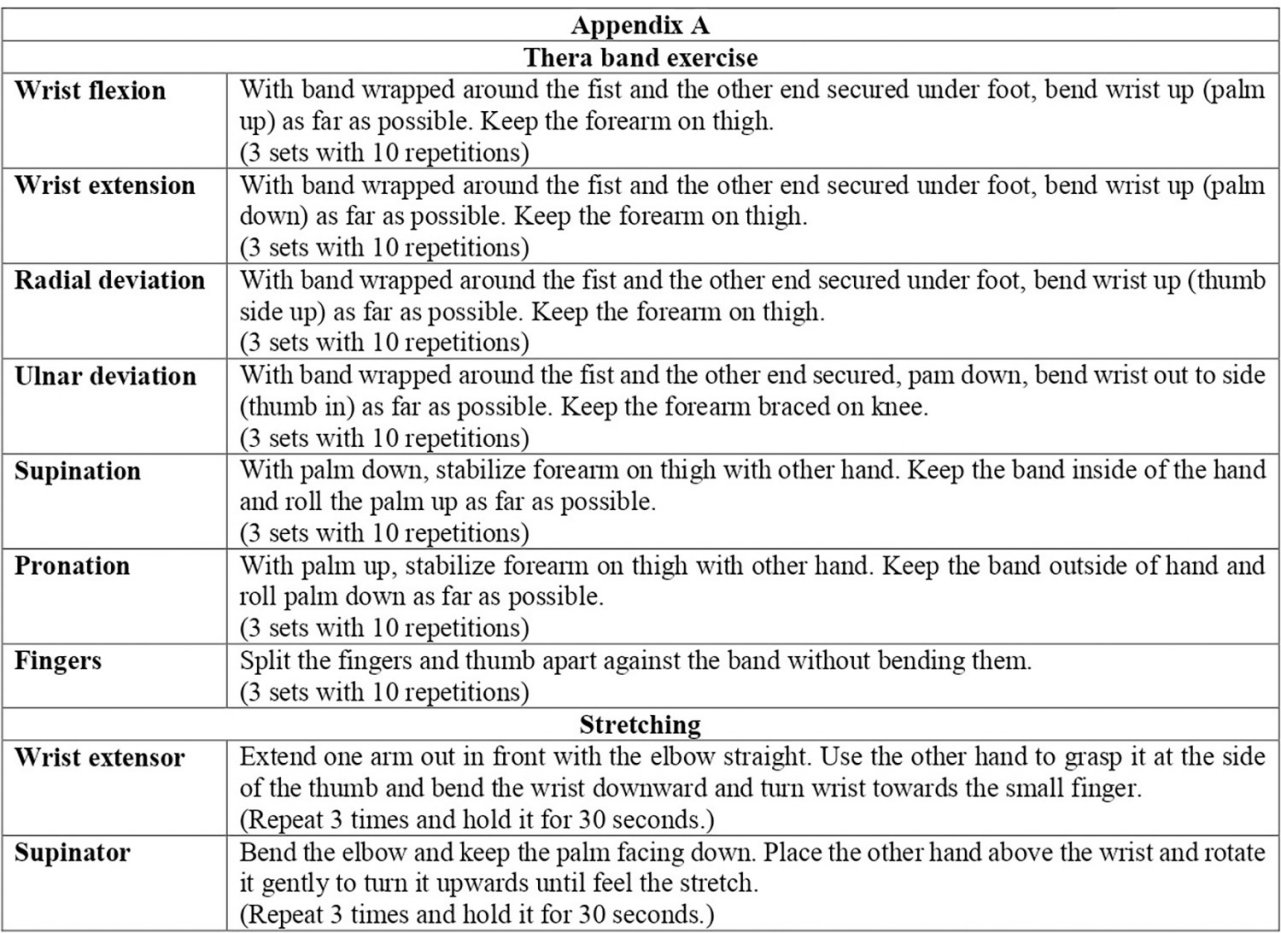
Appendix A–Theraband exercises.

Progressive resistance exercises (PRE) and home exercises were applied to both the treatment groups. The progressive resistance exercises were prescribed to the wrist joint with Thera-band based on the assessment of individual muscles, which was shown as an appendix-A (Fig 2). Initially, the painful movements are trained with minimal resistance and then progress to the next level of resistance for the other joint movements. In the later phase, the progression of exercise intends to work on activity specific rehabilitation. The therapist selected the exercise parameters (intensity, frequency and duration) in every treatment session purely based on the individual capacities without exaggerating the symptoms [17]. The participants were instructed to follow the correct form and posture to facilitate healing. The home exercises were performed daily for four weeks: consisting of eccentric exercise (3 times 30 repetitions) and isolated stretching of radial wrist extensors (3 times daily for 30 seconds). They were free from further treatment after four weeks of interventions. The treatment adherence was monitored by a treating therapist before the commencement of every session by checking the exercise log book.

### 2.4 Outcome measures

#### 2.4.1 Primary outcome

*2*.*4*.*1*.*1 Pain intensity*: It was measured with the visual analogue scale (VAS). The participant was asked to mark their pain intensity on the 10 cm point scale, where scores ranged from ’no pain’ (0) to ’ unimaginable pain’ (10). VAS is a valid and reliable tool to measure pain intensity [18].

#### 2.4.2 Secondary outcome

*2*.*4*.*2*.*1 Magnetic resonance imaging (MRI)*: It is an established assessment tool to measure the extent of common extensor tendon injury in LE patients. It was performed with a 3.0-T MR unit (Siemens Medical Solutions, Germany) with a flexible elbow coil (Philips, Nederland). T2 weighted axial and coronal sections were taken and the extent of the tear was classified as low (< 20%), intermediate (20–80%) and high grade (> 80%) according to the percentage of injury. It is a reliable and valid tool to measure percentage of injury in LE patients [13].

*2*.*4*.*2*.*2 Ultrasound (US) Imaging*: Ultrasound imaging was performed with a US unit (Esaote CA) with 8–18 MHz linear array transducer. Stages of LE was classified as: high grade tear (involves ≥ 50% of the common extensor tendon), low-grade tear (involves ≤ 50% of the common extensor tendon), suspected tendon tear (possible but not evident tear), no tendon tear [13].

The MRI and ultrasound measurements were done by a blinded radiologist, who was not involved in the study.

*2*.*4*.*2*.*3 Functional disability*: The Patient-rated Tennis Elbow Evaluation (PRTEE) questionnaire was used to measure the functional disability of the TE patients. The items are rated on a 11 point likert scale and the disability is rated from 0 –no disability to 100 –significant functional disability. It is a valid and reliable tool to measure functional disability in LE [19].

*2*.*4*.*2*.*4 Hand grip strength*: It was measured with a handheld dynamometer and it is a reliable and valid measurement. The participant sat in a relaxed position with the elbow flexed at 90° and pronated position. The participant pressed the dynamometer with maximum effort and the three measurements were taken and their average was used for analysis [15].

*2*.*4*.*2*.*5 Patient perception*: It was measured with the Global perceived improvement questionnaire consisting of a 6-point Likert scale. It is a reliable and valid tool to measure the patient perception related to LE [20].

*2*.*4*.*2*.*6 Kinesiophobia*: The Tampa Scale for Kinesiophobia–adjusted version (TSK-AV) was used to measure the status of fear of injury. The scale consists of 13 items marked on a 4 point likert scale, maximum score indicates more fear of injury and less score indicates less fear of injury [21].

*2*.*4*.*2*.*7 Depression*: The Hospital Anxiety and Depression Scale (HADS) was used to measure the depression status of LE patients. It consists of seven items each for depression and anxiety subscales. Scoring for each item ranges from 0 to 3, with 3 denoting highest anxiety or depression level. A total subscale score of >8 points out of a possible 21 denotes considerable symptoms of anxiety or depression [22].

*2*.*4*.*2*.*8 Quality of life*: The EuroQol EQ-5D was used to measure the health-related quality of life, expressed as utility values ranging from 1 to 3, where 1 represents perfect health [23].

### 2.5 Sample size

The sample size was calculated using a previous pilot study conducted in the department with primary outcome data of pain intensity measured with VAS scores. The ***required*** sample size was calculated by assuming 80% power with significance level of 0.05. To detect the minimum mean difference of 1.5 points and a standard deviation of 4, the sample size required was 27 in each group. When considering a 10% drop out, the samples required in each group was 30. For other outcomes, we considered a between-group difference of 30% of the outcome measure’s scale to be clinically worthwhile.

### 2.6 Blinding

Due to the design and settings of the study, it is not possible to blind the treating therapist involved in the study. The participants and the therapist who is assessing the outcomes at baseline, after 4 weeks, 8 weeks and at 6 months were blinded. Subjects were instructed not to disclose the study procedures and treatment protocol with fellow subjects and the assessing therapist.

### 2.7 Statistical analysis

The data analysis was performed by a statistician who did not participate in the recruitment, evaluation and treatment aspects of the study. The study homogeneity was analyzed using the Kolmogorov-Smirnov test. The data analysis was performed on an intention-to-treat basis. For the missing data, results obtained in the last available assessment of each participant were repeated. The time and group (4 × 2) of each outcome variable was analyzed with linear mixed model (LMM) analysis. The effects between the active and sham groups were analyzed by between group analysis and the intra group effects were analyzed by within group analysis for various intervals. The mean difference (MD) and 95% confidence interval (CI) were also calculated for each between-group comparison. The statistical analyses were processed using a commercial statistical software (IBM SPSS Statistics for Windows—version 20.0) and a level of significance of p≤0.05 was adopted for all tests.

## 3. Results

### 3.1 Flow of participants through the study

Flow of study participants throughout the trial is depicted in Fig 1 and their demographic characters (age, height, weight and duration of pain) were homogeneous as described in Table 1. A total of 115 participants were screened, 60 matched the selection criteria and were randomized into two groups. 5 participants dropped out during the 6 months follow up period and 55 participants completed the study period. The baseline values of the primary and secondary outcome measures were also similar and are presented in Table 2.

**Table 1:** Demographic and clinical characters of active and sham MM groups.

Sr. No	Variable	Active MM (n = 30)	Sham MM (n = 30)	p-value
1	Age (y)	48.35 ± 3.8	47.12 ± 3.5	0.197
2	Height (m)	1.67 ± 0.15	1.68 ± 0.16	0.803
3	Weight (kg)	71.8 ± 4.9	72.2 ± 4.7	0.748
4	Side involved (%)			
	Right side	25 (83%)	24 (80%)	-
	Left side	4 (13%)	6 (20%)	-
	Bilateral	1 (3%)	-	-
5	Dominance side affected (%)			
	Dominance = Right	20/27 (74%)	19/28 (68%)	-
	Dominance = Left	1/3 (33%)	1/2 (50%)	-
6	Previous episodes of LE, N (%)	6/30 (20%)	5/30 (17%)	-
7	Duration of pain (m)	6.2 ± 2.3	5.6 ± 2.7	0.358
8	Employment			
	Manual work	21/30 (70%)	20/30 (67%)	-
	Non-manual work	6/30 (20%)	6/30 (20%)	-
	Not working	3/30 (10%)	4/30 (13%)	-

MM–Mill’s manipulation, y–year, m–meter, kg–kilogram, LE–Lateral epicondylitis, m–months.

**Table 2:** Pre and post primary and secondary outcome measures of active and sham MM groups.

Sr. No	Variable	Duration	Active MM Group	Sham MM Group	Group × Time Effect size η^2^
1	Pain intensity–VAS (0–10 cm)	Base line	7.6 ± 1.4	7.4 ± 1.3	p = 0.001* η^2^ = 0.98
		4 weeks	3.8 ± 1.2	5.4 ± 1.2	
		8 weeks	1.6 ± 0.7	3.6 ± 1.1	
		6 months	0.9 ± 0.1	2.9 ± 0.9	
2	MRI T2 axial section—% (Percentage of injury)	Base line	58.5 ± 2.8	58.2 ± 2.7	p = 0.001* η^2^ = 0.99
		4 weeks	25.2 ± 1.9	43.2 ± 2.2	
		8 weeks	12.1 ± 0.9	31.2 ± 2.0	
		6 months	4.1 ± 0.5	20.1 ± 1.2	
3	MRI T2 coronal section—% (Percentage of injury)	Base line	61.7 ± 3.7	62.1 ± 3.8	p = 0.001* η^2^ = 0.96
		4 weeks	32.1 ± 2.4	44.2 ± 2.8	
		8 weeks	18.5 ± 1.6	33.1 ± 2.2	
		6 months	5.1 ± 0.5	20.6 ± 1.8	
4	Ultrasound Image—% (Percentage of injury)	Base line	60.5 ± 4.4	61.2 ± 4.3	p = 0.001* η^2^ = 0.93
		4 weeks	30.2 ± 3.1	42.6 ± 2.5	
		8 weeks	15.2 ± 1.3	29.8 ± 2.2	
		6 months	4.2 ± 0.4	19.5 ± 1.3	
5	Functional disability PRTEE (0–100)	Base line	74.1 ± 5.1	74.8 ± 4.9	p = 0.001* η^2^ = 0.90
		4 weeks	45.2 ± 3.2	58.1 ± 3.9	
		8 weeks	26.3 ± 1.6	46.2 ± 2.6	
		6 months	10.1 ± 0.9	27.3 ± 1.4	
6	Handgrip strength Hand dynamometer (kg)	Base line	29.2 ± 2.5	29.5 ± 2.6	p = 0.001* η^2^ = 0.55
		4 weeks	31.6 ± 2.6	29.9 ± 2.6	
		8 weeks	33.7 ± 2.6	31.9 ± 2.6	
		6 months	37.9 ± 2.7	32.5 ± 2.7	
7	Patient perception (GPI questionnaire)	Base line	1.3 ± 0.2	1.2 ± 0.2	p = 0.001* η^2^ = 0.84
		4 weeks	3.6 ± 0.3	2.2 ± 0.2	
		8 weeks	4.7 ± 0.4	2.8 ± 0.3	
		6 months	6.5 ± 0.6	3.4 ± 0.4	
8	Kinesiophobia (TSK-AV)	Base line	47.2 ± 3.6	46.8 ± 3.2	p = 0.001* η^2^ = 0.77
		4 weeks	28.1 ± 2.6	36.2 ± 2.8	
		8 weeks	20.2 ± 3.5	26.3 ± 2.2	
		6 months	6.5 ± 1.1	15.8 ± 1.9	
9	Depression (HADS)	Base line	15.8 ± 1.6	16.1 ± 1.5	p = 0.001* η^2^ = 0.82
		4 weeks	10.9 ± 1.4	14.2 ± 1.4	
		8 weeks	7.3 ± 0.8	10.2 ± 1.1	
		6 months	3.1 ± 0.4	8.9 ± 0.8	
10	Quality of life (EuroQol EQ-5D)	Base line	2.6 ± 0.7	2.5 ± 0.8	p = 0.001* η^2^ = 0.19
		4 weeks	2.0 ± 0.5	2.4 ± 0.5	
		8 weeks	1.4 ± 0.3	2.3 ± 0.3	
		6 months	1.1 ± 0.1	2.2 ± 0.2	

*Significant, MM–Mill’s manipulation

VAS–Visual analog scale, MRI–Magnetic resonance imaging, PRTEE—Patient-rated Tennis Elbow Evaluation, kg–Kilogram, GPI–Global perceived improvement, TSK–AV—The Tampa Scale for Kinesiophobia–adjusted version, HADS—Hospital Anxiety and Depression Scale, EuroQol EQ-5D –European quality of life–five dimension.

### 3.2 Compliance with the study protocol

All registered outcome measures are reported in this manuscript. There was minimal loss to follow-up, as presented in Fig 1. At 8 weeks and 6 months follow up, 90% and 93% of the participants provided follow-up data, respectively. Five participants did not complete the follow up measurement: three in the active MM group (two due to increase in pain and the one due to personal reason); two in the sham MM group (one due to personal reason and the one with time restriction).

### 3.3 Effects of the intervention

#### 3.3.1 Primary and secondary outcomes

The time and group (4 × 2) linear mixed model (LMM) of primary variable (pain intensity—VAS) reports statistically significant difference (p<0.001) between active and sham groups at baseline, 4 weeks, 8 weeks and at 6 months follow up. The post intervention at 4 weeks 1.6 (CI 95% 0.97 to 2.22) shows improvement in the active group than sham group. The similar effects have been noted after 8 weeks and at 6 months 2.0 (CI 95% 1.66 to 2.33) follow up. The scores show significant changes (p<0.001) in the active group than sham group which is presented in Table 2. The effect size of pain intensity (η^2^ = 0.98) shows a larger effect in the active MM group than sham MM group.

**Fig 3:**
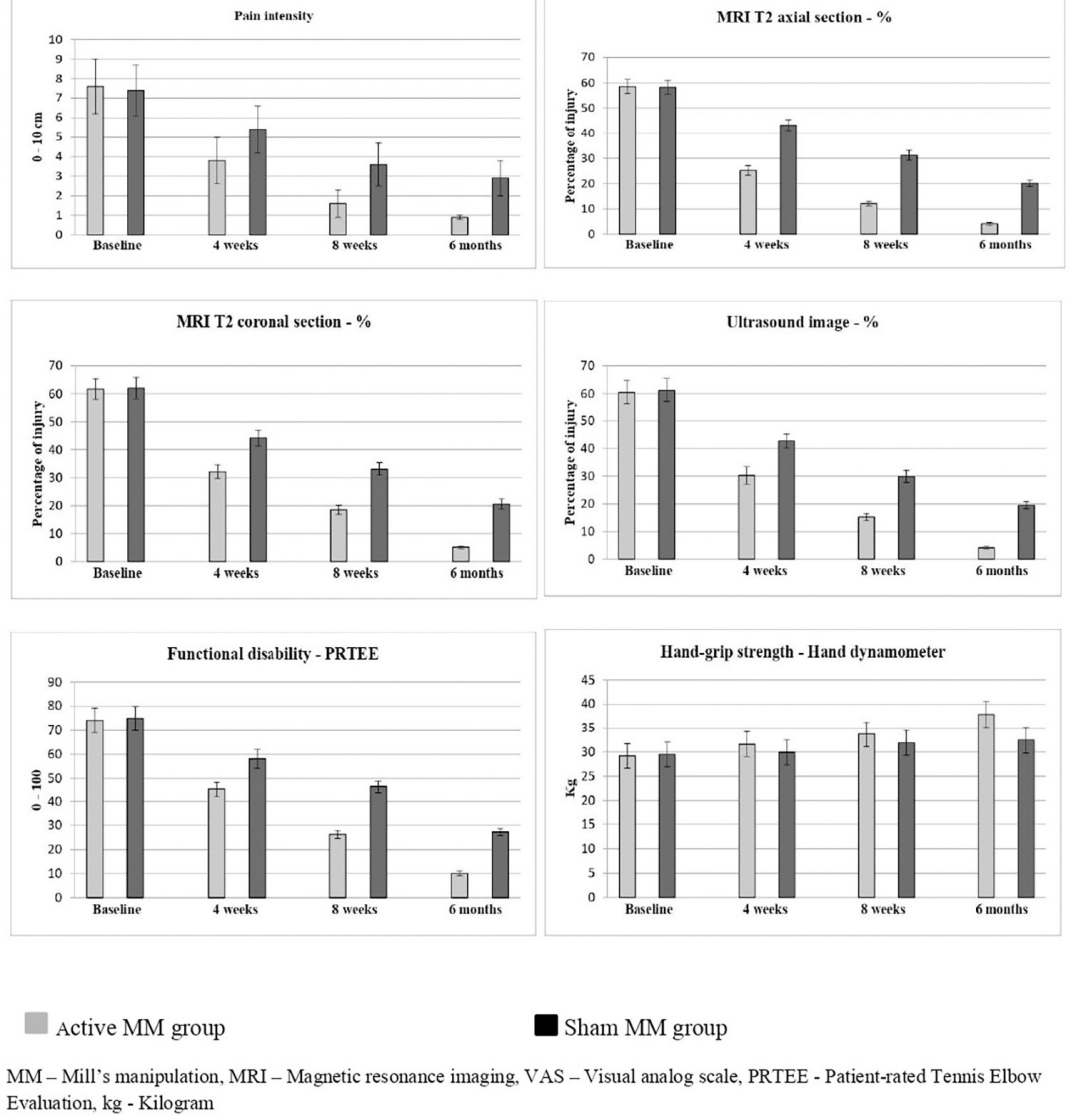
Pre and post primary outcome measures of active and sham MM groups.

The time and group (4 × 2) linear mixed model (LMM) of other variables (MRI T2 axial and coronal section, Ultrasound, Functional disability and Handgrip strength) report statistically significant difference (p = 0.001) between group A and group B at baseline, 4 weeks, 8 weeks and at 6 months follow up. The post intervention measure of percentage of injury through MRI and US image reports statistical change (p = 0.001) at 4 weeks’ duration. The similar effects were noted after 8 weeks and at 6 months follow up measurement, which is shown in Table 2 and Fig 3. The same changes have been noted in the functional disability and handgrip strength at 4 weeks, 8 weeks and at 6 months follow up. The effect size of MRI T2 axial (η^2^ = 0.99), MRI T2 coronal CSA (η^2^ = 0.96), Ultrasound (η^2^ = 0.93) functional disability (η^2^ = 0.90) and handgrip strength (η^2^ = 0.55) shows larger effect in active MM group than the sham MM group.

**Fig 4:**
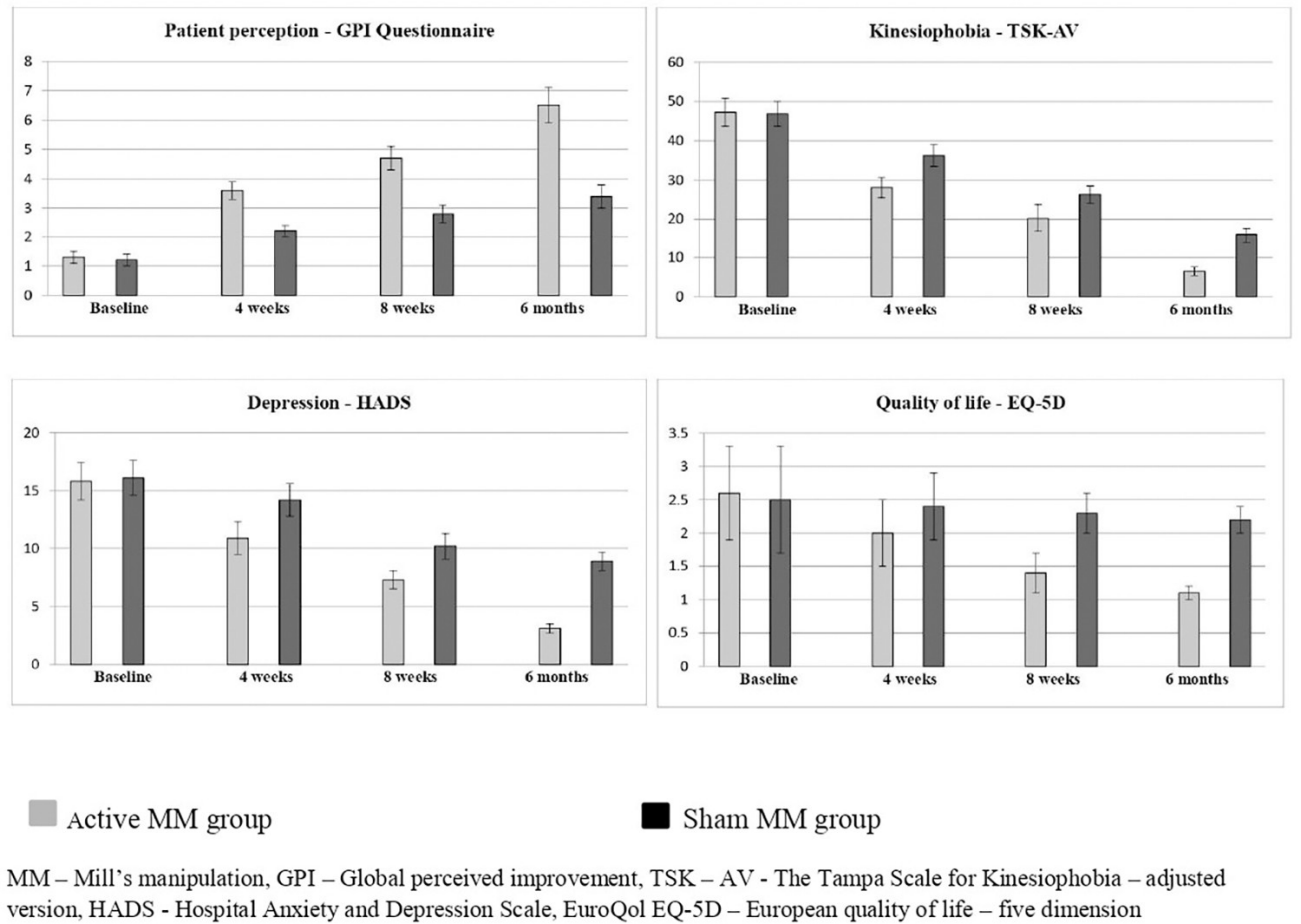
Pre and post-secondary outcome measures of active and sham ESWT groups.

The time and group (4 × 2) linear mixed model (LMM) of all the other variables (patient perception, kinesiophobia, depression and quality of life) reports statistically significant difference (p = 0.001) between active and sham groups at baseline, 4 weeks, 8 weeks and at 6 months follow up. The post intervention at 4 weeks of all secondary variables shows improvement in the active group than the sham group. The similar effects have been noted after 8 weeks and at 6 months follow up. The scores show significant changes (p = 0.001) in the active group than the sham group which is shown in Table 2. For the primary and secondary outcome measures, important between-group differences were detected. The between-group differences had positive effects, which were clinically worthwhile. The confidence intervals around these estimates of the treatment effect should be generally included and is depicted in Table 3. The Figs 3 and 4 also shows more improvements in all the variables in the active MM group than the sham MM group at various intervals.

**Table 3:** Pre and post mean difference and confidence interval (upper limit and lower limit) of primary and secondary outcome scores of active and sham MM groups.

		Mean difference CI 95% (Upper limit–Lower limit)		
		4 weeks	8 weeks	6 months
Pain intensity	Active × Sham	1.6 (0.97 to 2.22)	2.0 (1.49 to 2.50)	2.0 (1.66 to 2.33)
	p–value	0.001	0.001	0.001
MRI—T2 axial	Active × Sham	18.0 (16.93 to 19.06)	19.0 (18.29 to 19.90)	16.0 (15.55 to 16.44)
	p–value	0.001	0.001	0.001
MRI—T2 coronal	Active × Sham	12.1 (10.75 to 13.44)	14.6 (13.60 to 15.59)	15.5 (15.05 to 15,94)
	p–value	0.001	0.001	0.001
Ultrasound Image	Active × Sham	12.4 (10.94 to 13.85)	14.6 (13.66 to 15.33)	15.3 (14.82 to 15.77)
	p–value	0.001	0.001	0.001
Functional disability	Active × Sham	12.9 (11.05 to 14.74)	19.9 (18.78 to 21.01)	17.2 (16.59 to 17.80)
	p–value	0.001	0.001	0.001
Handgrip strength	Active × Sham	-1.7 (-3.04 to -0.35)	-1.8 (-3.14 to -0.45)	-5.4 (-6.79 to -4.00)
	p–value	0.014	0.009	0.001
Patient perception	Active × Sham	-1.4 (-1.53 to -1.26)	-1.9 (-2.08 to -1.71)	-3.1 (-3.36 to -2.83)
	p–value	0.001	0.001	0.001
Kinesiophobia	Active × Sham	8.1 (6.70 to 9.49)	6.1 (4.58 to 7.61)	9.3 (8.49 to 10.10)
	p–value	0.001	0.001	0.001
Depression	Active × Sham	3.3 (2.57 to 4.02)	2.9 (2.40 to 3.39)	5.8 (5.47 to 6.12)
	p–value	0.001	0.001	0.001
Quality of life	Active × Sham	0.4 (0.14 to 0.65)	0.9 (0.74 to 1.05)	1.1 (1.01 to 1.18)
	p–value	0.003	0.001	0.001

### 3.4 Adverse events with the study intervention

There was no adverse reactions or side effects noted in both the experimental and control groups during and after the corticosteroid injection and joint manipulation treatment.

## 4. Discussion

Participants in the active group showed significant improvement compared to the sham group at various intervals in all the outcome measures. Sham group also showed a statistically significant improvement over time across the primary and secondary outcomes. A study by Olaussen M et al, observed that corticosteroid (CS) injection with physiotherapy has very good initial response in chronic LE [24]. Our reports are in agreement with Olaussen M et al and also found an added effect of DTFM and Mill’s manipulation (MM) in physical therapy in LE. On the contrary, Coombes et al observed that combination of CS injection and physiotherapy showed no added benefit in LE on a long-term basis. They opposed the use CS due to high recurrence rates and adverse reaction at the later stage [25]. CS injection provides considerable pain reduction soon after the administration, which promotes an excessive use of the limb at the earlier stage [26]. This is one of the main reasons for the high relapse rates at the later stage of steroid injection. Therefore, the patients are instructed to keep the limb in a resting state for 2 days after the injection. Also, the anesthetic agent applied has synergistic action with the CS and extends the power of action making the intervention more attractive to the patient [27].

The therapeutically effective deep transverse friction massage with Mill’s manipulation provided in this study, consists of three sessions per week for four weeks’ which was considered as suitable for treating chronic LE. However, there was insufficient clinical evidence for the manual therapy alone in a long-term basis [28]. Stasinopoulos D et al observed that Mill’s manipulation performed immediately after the deep transverse friction massage (DTFM) would provide a full range of motion at the elbow joint [29]. DTFM attempts to prevent or destroy abnormal fibrous adhesions (cross‐links or cross‐bridges) by imposing stress transversely to the remodeling collagen of the tissue to soften the adhesion. It also optimizes the quality of the scar tissue by realigning the collagen of normal soft tissue fibers in a longitudinal way and enhances normal healing conditions and prevents abnormal scarring [30]. Kushner S et al stated that Mill’s manipulation facilitates the elongation of the tight fibrous tissues by rupturing adhesions within the teno-oseous junction. This maneuver makes the area mobile, pain free and considerably improves the wrist function. They affirm that manipulation apparently initiates a chain reaction, restoring the physiological function of the affected tissues [31]. Our findings also confirm that DTFM and Mill’s manipulation with progressive resistance exercise would be useful for improving the condition.

Our study, measured the extent and percentage of injury through MRI and US and found that active group has significant improvement than sham group. Coel et al. found that the percentage of injury would correlate with abnormal motion or compensation caused by injury related pain [32]. There were persistent and significant changes in pain intensity scores from baseline, each follow-up time points for both the groups. The pattern of improvement identified in our study suggests that progressive resistance exercise offered more rapid improvement. Increase in muscle strength improves the functional status [15] and psychological (patient perception, kinesiophobia, depression and QOL) status of the participants. Use of a sham maneuver combined with blinding is intended to prevent bias resulting from non-specific effects associated with those receiving the intervention (Hawthorne effect) [33]. A low velocity and high amplitude technique was used as a sham comparison in sham group, however the “Hawthorne effect” may be responsible for the clinical changes.

### 4.1 Strengths and weaknesses

We used 8 weeks and 6 months’ follow-up with several outcomes to register the development of improvement after different interventions. The clinical setting and the choice of ordinary, well known physiotherapeutic approach ensured the external validity. This study strictly adhered to the CONSORT guidelines for randomized controlled trials. Randomization, concealed allocation and blinding strategies were performed, as these features are known to minimize bias. There were a few dropouts and the adherence to the intervention was good. We chose the apt statistical tests that would minimize Type-1 errors, since this is a non-serious and self-limiting condition. Only male subjects were included in the study. Including both genders may provide information about these interventions for both sexes and can generalize the results. A strict physiotherapy guideline, prohibited an individual adjustment of treatment and may have influenced the results. Although much research could be done on Mill’s manipulation alone, it would be interesting to study the efficacy on acute and sub-acute conditions, using similar inclusion criteria as in our study.

## 5. Conclusion

Corticosteroid injection with deep transverse friction massage and Mill’s manipulation is effective in terms of reducing pain, improving tissue healing, functional disability, handgrip strength, patient perception, kinesiophobia and depression status and, health related quality of life in people with lateral epicondylalgia.

## Supporting information

S1 Checklist CONSORT 2010 checklist of information to include when reporting a randomised trial*. (RTF)

S1 Data (XLSX)

S1 File (DOCX)
