# Supplementary material for: Clinical and radiological effects of Corticosteroid injection combined with deep transverse friction massage and Mill’s manipulation in lateral epicondylalgia–A prospective, randomized, single-blinded, sham controlled trial
Source: PLoS One. 2023 Feb 13;18(2):e0281206. doi: 10.1371/journal.pone.0281206 (PMC9925075; doi:10.1371/journal.pone.0281206)
Supplement: S1 File — (DOCX) [file pone.0281206.s003.docx]

**Title:** Clinical and radiological effects of Corticosteroid injection combined with Mill’s manipulation in Lateral epicondylitis – A prospective, randomized, single-blinded, sham controlled trial.

Gopal Nambi**^1^,** Mshari Alghadier^1^, Anju Verma^1,2^, Osama R. Aldhafian^3^, Naif A. Nwihadh^4^**,** Ayman K. Saleh^3,5^ Mohamed A. Omar^5^, Tohamy G.T. Hassan^5^, Mohamed Nagah Ahmed Ibrahim^5^, Hassan Fathy El Behairy^6^

**^1^** Department of Health and Rehabilitation Sciences, College of Applied Medical Sciences, Prince Sattam bin Abdulaziz University, Al kharj, Saudi Arabia.

^2^ Department of Exercise and sports, University of Sydney, New South Wales, Australia.

**^3^**Department of Surgery, College of Medicine, Prince Sattam Bin Abdulaziz University, Al Kharj, Saudi Arabia.

^4^ King Fahad medical city, Riyadh, Saudi Arabia.

**^5^** Department of Orthopedic Surgery, Faculty of Medicine for Girls, Al-Azhar University, Cairo, Egypt.

^6^ Al-zhraa University Hospital, Faculty of Medicine for Girls, Al-Azhar University, Cairo, Egypt.

**Clinical trial registration number:** CTRI/2020/04/025135 registered prospectively on 12/05/2020.

**Study Protocol**

**Background:** Chronic lateral epicondylitis is the major health concern and common consequence of repetitive work in the common population. Moreover, this clinical condition has not been considered in usual physical rehabilitation practice and nor its rehabilitation part is well defined, which requires a meaningful study in this field.

**Objective:** To find and compare the effects of corticosteroid injection (CS) and Mill’s manipulation on radiological changes in chronic lateral epicondylitis.

**Design:** It is a single-blinded, randomized, experimental study performed during 1^st^ June 2020 and 1^st^ August 2021. The trial received acceptance from the department of ethical committee (DEC), Prince Sattam bin Abdulaziz University, Al-Kharj, Saudi Arabia with an approval number of RHPT/020/012. The DEC approved the subject consent form, treatment protocols and the outcome parameters measured in the trial. The trial was executed in accordance with the ethical guidelines laid down in the 1964 Declaration of Helsinki and was registered in the clinical trial registry with registration number CTRI/2020/04/025135 on 12/05/2020. The finally selected subjects for the trial were asked to fill the written subject consent form and underwent measurements for pre interventional personal and anthropometric data. The participants were randomized using computer generated randomization method and allocated into two groups: corticosteroid injection with Mill’s manipulation (MM) – Active group (n=30) and corticosteroid injection with Mill’s manipulation (MM) - Placebo group (n = 30).

***Subjects***

Subjects for the study were received from local and government hospitals in Al-Kharj and Riyadh region of Saudi Arabia. All the participants were informed about the harms and benefits of the research through an information form. Subjects who consented to participate were selected for the study. Male, aged between 18 and 60 years, with a clinical diagnosis of chronic (> 2 months of pain) lateral epicondylitis (M77.1 in ICD-10 – International classification of diseases), with pain intensity between 3 to 8 on the visual analog scale (VAS) were invited to participate. Participants with prior steroid injection therapy, associated neck or arm pain, severe musculoskeletal, neural, somatic and psychiatric conditions, waiting for surgery, having alcohol or drug abuse, involving in other weight training programs, and red flags to manipulation were excluded from the study. Participants with other soft tissue injuries, fracture at the limbs, deformities were also excluded from the study. The fig-1 depicts the methods and procedure of involving study subjects in this trial.

***Intervention***

Following corticosteroid injection, the recommended physical therapy was given for 4 weeks, after which the participants were asked to exercise at home for another 4 weeks. The participants maintained an exercise log book during the study period.

Corticosteroid injection

A physical examination was performed by an orthopedic surgeon before the administration of injection. 1 ml Triamcinolone Acetonide (10 mg/ml) (Kenacort- A 10) with 1 ml Lignocaine (1%) was administered into the most palpably tender point in the region of the lateral epicondyle.^15^ To maintain participant blinding, the participants were not allowed to see the procedure of administration of injection. In addition, post-injection instructions were given to all the participants. They were asked to take rest and not engage in strenuous activities for one week following injection, even if they experience pain relief. Any adverse consequences were noted and treated by the prime investigator.

Physical therapy

A week after the injection the participants started with the physical therapy interventions. All participants received the respective physical therapy for 3 sessions per week for 4 weeks, each session lasted for 30 to 40 minutes.

All the participants underwent baseline evaluation of variables before the administration of injection. To avoid intervention bias, a fixed physical therapy protocol was prepared on the basis of recent evidence with the objectives of ameliorating pain, increasing functional activity and soft tissue healing. A holistic approach consisting of physical modalities, exercise protocols and patient education were used to obtain these objectives. Participants in active MM group underwent Mill’s manipulation, in which the participant was asked to sit comfortably with proper backrest. The treating therapist stands behind the patient and grasp the patient’s arm under the crook of the elbow with the shoulder joint abducted to 90° and medially rotated. Therefore, the patient’s forearm is pronated. Place the thumb of other hand in the web space between the patient’s thumb and index finger and fully flex the patient’s wrist and pronate the forearm. Move the hand supporting the crook of the elbow on to the posterior surface of the elbow joint and, while maintaining full wrist flexion and pronation, extend the patient’s elbow until that all the slack has been taken up in the tendon. Apply a high velocity low amplitude thrust (HVLAT) by side flexing the body away from the arms and pushing downwards with the hand over the patient’s elbow. This procedure is conducted only once at each session because it is an uncomfortable procedure for the patient^.16^ For the placebo group, low velocity high amplitude movement was performed at the elbow joint, which was not comprehensible to the study participants.

Progressive resistance exercises (PRE) were prescribed for the wrist extensors with Thera-band based on the assessment of individual muscles. Initially, the painful movements are trained with minimal resistance and then progress to the next level of resistance for the other joint movements. In the later phase, the progression of exercise intends to work on activity specific rehabilitation. The therapist selected the exercise parameters (intensity, frequency and duration) in every treatment session purely based on the individual capacities without exaggerating the symptoms.^17^ The participants were instructed to follow the correct form and posture to facilitate healing. The home exercises were performed daily for four weeks: consisting of eccentric exercise (3 times 30 repetitions) and isolated stretching of radial wrist extensors (3 times daily for 30 seconds). The treatment adherence was monitored by a treating therapist before the commencement of every session by checking the exercise log book.

***Outcome measures***

Pain intensity: It was measured with the visual analogue scale (VAS). The participant was asked to mark their pain intensity on the 10 cm point scale, where scores ranged from 'no pain' (0) to ' unimaginable pain' (10). VAS is a valid and reliable tool to measure pain intensity.^18^

Magnetic resonance imaging (MRI): It is an established assessment tool to measure the extent of injury in LE patients. It was performed with a 3.0-T MR unit (Siemens Medical Solutions, Germany) with a flexible elbow coil (Philips, Nederland). T2 weighted axial and coronal sections were taken and the extent of the tear was classified as low (< 20%), intermediate (20–80%) and high grade (> 80%) according to the percentage of injury.^13^

Ultrasound (US) Imaging: Ultrasound imaging was performed with a US unit (Esaote CA) with 8-18 MHz linear array transducer. Stages of LE was classified as: high grade tear (involves ≥ 50% of the tendon), low-grade tear (involves $\leq$50% of the tendon), suspected tendon tear (possible but not evident tear), no tendon tear.^13^

Functional disability: The Patient-rated Tennis Elbow Evaluation (PRTEE) questionnaire was used to measure the functional disability of the TE patients. The items are rated on a 11 point likert scale and the disability is rated from 0 – no disability to 100 – significant functional disability. It is a valid and reliable tool to measure functional disability in LE.^19^

Hand grip strength: It was measured with a handheld dynamometer and it is a reliable and valid measurement. The participant sat in a relaxed position with the elbow flexed at 90^o^ and pronated position. The participant pressed the dynamometer with maximum effort and the three measurements were taken and their average was used for analysis.^15^

Patient perception: It was measured with the Global perceived improvement questionnaire consisting of a 6-point Likert scale. It is a reliable and valid tool to measure the patient perception related to LE.^20^

Kinesiophobia: The Tampa Scale for Kinesiophobia – adjusted version (TSK-AV) was used to measure the status of fear of injury. The scale consists of 13 items marked on a 4 point likert scale, maximum score indicates more fear of injury and less score indicates less fear of injury.^21^

Depression: The Hospital Anxiety and Depression Scale (HADS) was used to measure the depression status of LE patients. It consists of seven items each for depression and anxiety subscales. Scoring for each item ranges from 0 to 3, with 3 denoting highest anxiety or depression level. A total subscale score of >8 points out of a possible 21 denotes considerable symptoms of anxiety or depression.^22^

Quality of life: The EuroQol EQ-5D was used to measure the health-related quality of life, expressed as utility values ranging from 1 to 3, where 1 represents perfect health.^23^

***Sample size***

With a power of 0.8 and a significance level of 0.05, at least 30 participants were needed in each treatment arm (60 participants in total) to detect a clinically important mean difference between groups of 4 points on the VAS scores at 6 months, when assuming a standard deviation of 1 point and considering a 10% drop to follow-up. For other outcomes, we considered a between-group difference of 20% of the outcome measure’s scale to be clinically worthwhile.

**Statistical analysis plan**

The data analysis was performed by a statistician who did not participate in the recruitment, evaluation and treatment aspects of the study. The study homogeneity was analyzed using the Kolmogorov-Smirnov test. The data analysis was performed on an intention-to-treat basis. For the missing data, results obtained in the last available assessment of each participant were repeated. Analysis of variance with a linear mixed model was used to compare the effects of corticosteroid injection with MM between the active and placebo groups. The mean difference (MD) and 95% confidence interval (CI) were also calculated for each between-group comparison. The statistical analyses were processed using a commercial statistical software and a level of significance of p≤0.05 was adopted for all tests.

**Informed Consent Form**

Name of patient: Age/Gender:

**Study Title:** “Clinical and radiological effects of Corticosteroid injection combined with Mill’s manipulation in Lateral epicondylitis – A prospective, randomized, single-blinded, sham controlled trial.”

**Introduction and Purpose of Study:** You have been selected to take part in a study “MRI and Ultra sound analysis of Corticosteroid injection combined with Mill’s manipulation in Lateral epicondylitis – A prospective, randomized, double-blinded, placebo controlled trial”

**Study Information**

**Outline of Procedures:** At first consultation you will be screened and evaluated for suitability of the study. You are requested to attend 4 sessions a week for 8 weeks’ period. If you are taking any medication or undergoing any other form of treatment for your pain, you may be excluded from the study.

**Risks and Discomfort:** The treatment is safe and is unlikely to cause any adverse side effects. All treatments will be performed by qualified physiotherapist.

**Benefits of the study:** This study will assist the physiotherapy profession in expanding its knowledge of this condition and thus making future treatment of patients suffering from chronic low back pain more effective.

**Withdrawal from the Study:** You are free to withdraw at any stage with no negative repercussions to your health care.

**Remuneration:** Patients taking part in the study will not be offered any other form of remuneration for taking part in the study.

**Costs of Study:** Treatment for the duration of the research process will be free of charge. **Confidentiality:** All patient information and results will be kept confidential and can be shared for research purpose if required.

I have been explained about this research in which I agreed to participate. I know that I am giving this consent without any force. I can discontinue the study any time without any reason and that will not affect my treatment that I have been informed. My identity can be disclosed for any other follow up research.

**Signature of Patient Signature of Researcher**

I certify that I have explained to the participant about nature, purpose, potential benefits & possible risk of the indicated procedure. The information collected will be kept confidential.

**Signature of Witness Signature of Researcher**

**DECLARATIONS**

**Ethics approval and consent to participate:** The study obtained the study ethical approval from Department Ethical Committee (DEC), Prince Sattam bin Abdulaziz University, Al-Kharj, Saudi Arabia with the reference number of RHPT/020/012. Written consent was obtained from all the participants who involved in the study.

**Clinical trial registration:** CTRI/2020/04/025135 registered prospectively on 12/05/2020.

**Consent for publication:** No individual data is contained in this publication.

**Availability of data and materials:** Data is not publically available, but can be obtained from the corresponding author on request.

**Competing interest:** None declared

**Funding:** Self-funded study.

**Acknowledgement:** This research was supported by the Deanship of scientific research, Prince Sattam bin Abdulaziz University, Saudi Arabia.
